# Supplementary material for: Minimally sufficient experimental design using identifiability analysis
Source: NPJ Syst Biol Appl. 2024 Jan 6;10:2. doi: 10.1038/s41540-023-00325-1 (PMC10771435; doi:10.1038/s41540-023-00325-1)
Supplement: Supplementary file 1 — Supplementary Information [file 41540_2023_325_MOESM1_ESM.pdf]

Minimally Sufficient Experimental Design using Identifiability Analysis

Jana L. Gevertz and Irina Kareva

Supplementary Information

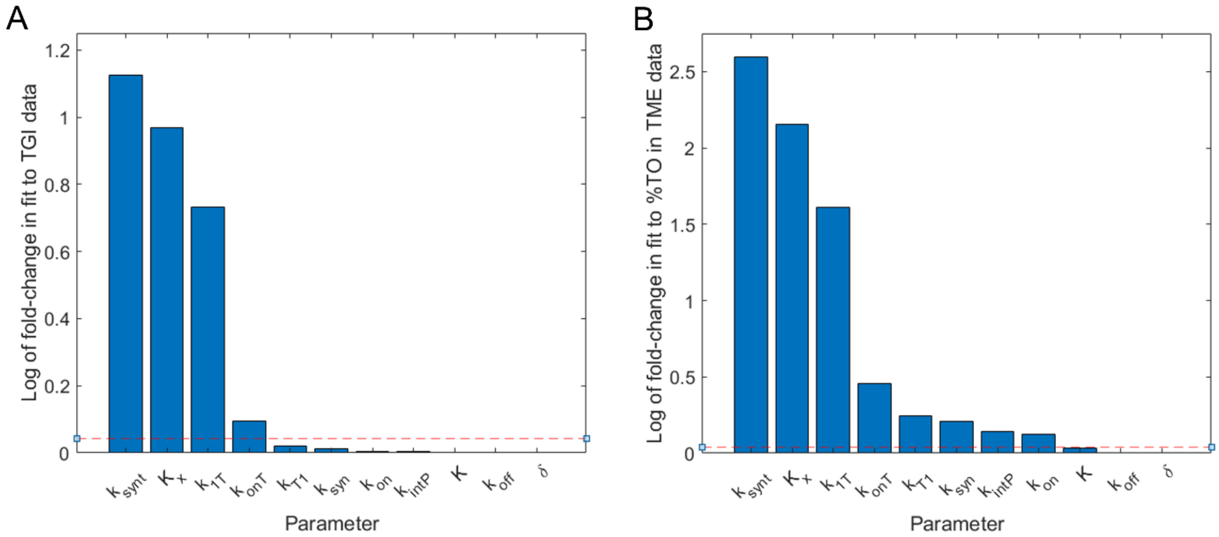

Supplementary Figure 1: Local sensitivity analysis of a subset of model parameters. Each parameter was individually varied by $\pm 10\%$, and the maximum of the log of the fold-change in model fit (relative to the best fit parameter set) was computed. (A) Sensitivity of fit to TGI data. (B) Sensitivity of fit to %TO in the TME data. The dashed line indicates the fold-change in the cost function that is equal to the fold-change in the parameter value.

Supplementary Table 1: Computational resources and costs associated with various steps of the proposed minimal experimental design workflow. Note that, unless otherwise noted, all profiles here were generated by using 50 points uniformly distributed over each parameter's domain.

Code	Where/how ran	Run time	How to speed up run time
Fit model to simulated %TO in TME data and generate parameter profiles using all simulated data	Locally, not parallelized	@21min	N/A (Only used 12 points per profile)
Generate parameter profiles using every possible 1-day protocol (30 such protocols)	Locally, and parallelized to run over 4 "tasks"	@4.5h	1. Use less than 50 points per profile<br>2. Use only a subset of 1-day protocols (ex: test every other day)<br>3. Parallelize over more "tasks"
Generate parameter profiles using every possible 2-day protocol (435 such protocols)	High performance cluster, parallelized over 63 "tasks"	@6.8h	1. Use less than 50 points per profile<br>2. Use only a subset of 2-day protocols (a random N%)
Generate parameter profiles using a random 1/3 of all possible 3-day protocols and removing non-unique samples (tested 1126 of 4060 protocols)	High performance cluster, parallelized over 63 "tasks"	@16.7h	1. Use less than 50 points per profile<br>2. Use less than 33% of all 3-day protocols

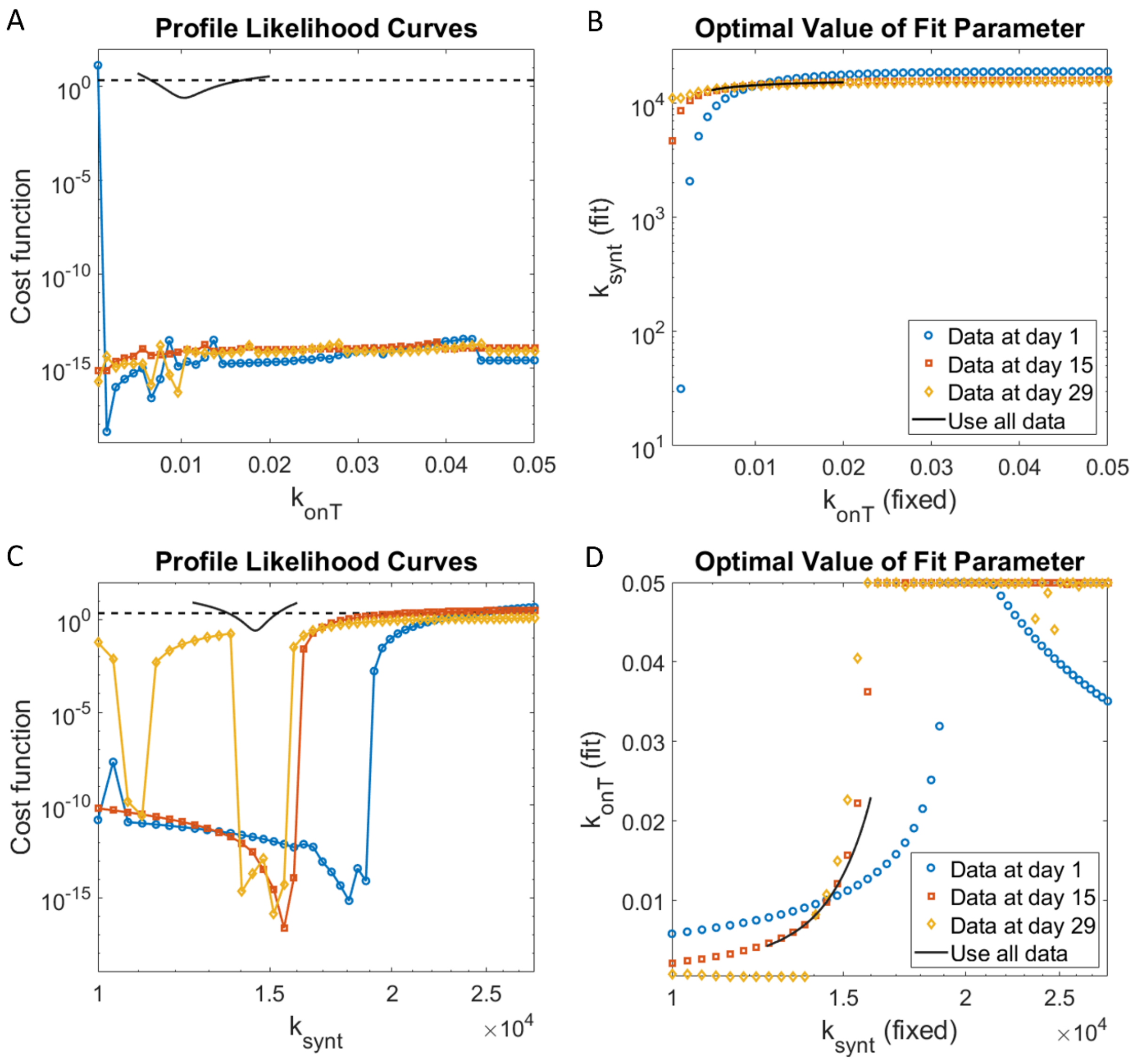

Supplementary Figure 2: Profile likelihood curves and corresponding best-fit parameters when percent target occupancy is measured early, mid, or late month. Profile likelihood curves are shown for (A) k_{onT} and (C) k_{synt} when percent target occupancy in the TME is collected only on day 1 (blue circle), day 15 (red square), or day 29 (orange diamond). (B) shows the best-fit value of k_{synt} for each fixed value of k_{onT}. (D) shows the best-fit value of k_{onT} for each fixed value of k_{synt}. Black solid lines represent results using complete data (daily measurement of %TO in TME), and the black dashed line is the 95% confidence threshold for the profiled parameter using all complete data. Any parameter whose cost falls below the 95% confidence threshold, paired with the corresponding best-fit value of the fit parameter, form a “plausible” parameter set that is considered in Figure 6. It is of note that we restrict $0.0005 \le k_{onT} \le 0.05$ when performing parameter fitting.

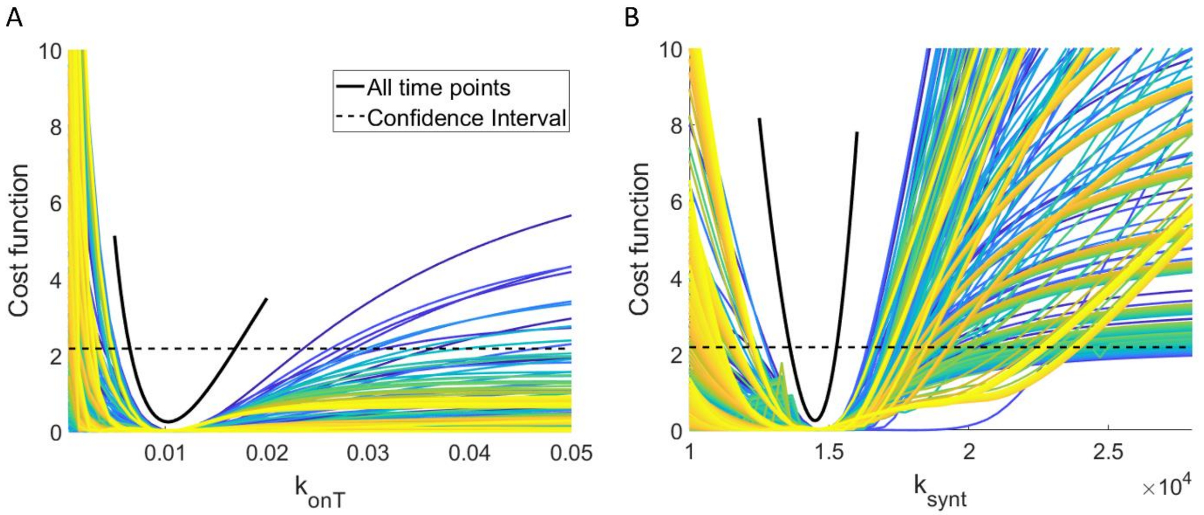

Supplementary Figure 3: Profile likelihood curves for all 2-day experimental protocols. For all 435 protocols that collect percent target occupancy in the TME at exactly two days, the profile likelihood curves are shown for (A) k_{onT} and (B) k_{synt}. The color indicates the spacing between collection days, ranging from spacing the experiments by one day (blue) to 29 days (yellow). The solid black curve is the profile likelihood curve for the specified parameter when complete data is used, and the black dashed line is the corresponding 95% confidence threshold.

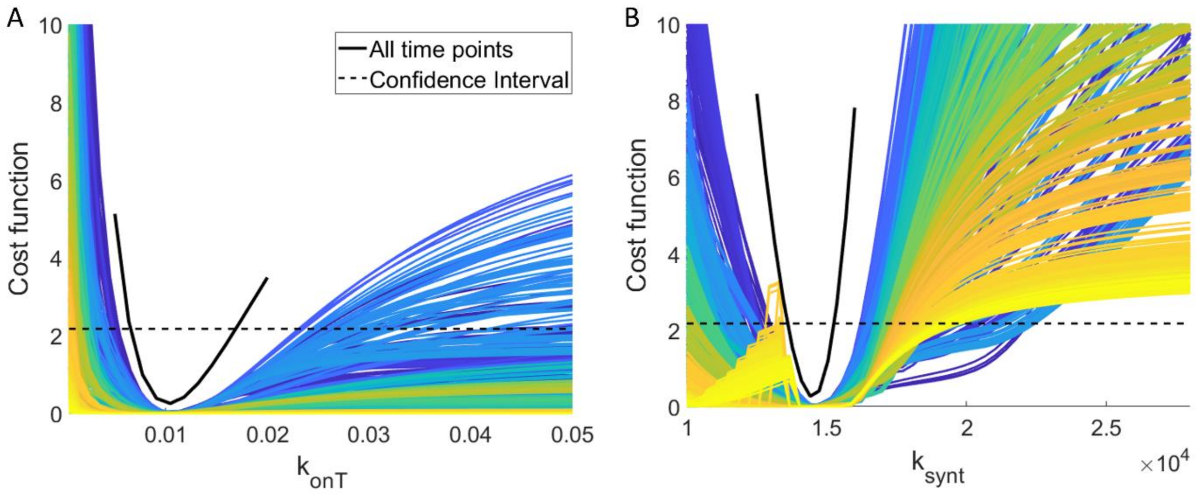

Supplementary Figure 4: Profile likelihood curves for a subset of 3-day experimental protocols. For a randomly-sampled set of 1126 protocols that collect percent target occupancy in the TME at exactly three days, the profile likelihood curves are shown for (A) k_{onT} and (B) k_{synt}. The color indicates the spacing between the first two collection days, ranging from spacing the first two experiments by one day (blue) to 28 days (yellow). The solid black curve is the profile likelihood curve for the specified parameter when complete data are used, and the black dashed line is the corresponding 95% confidence threshold.

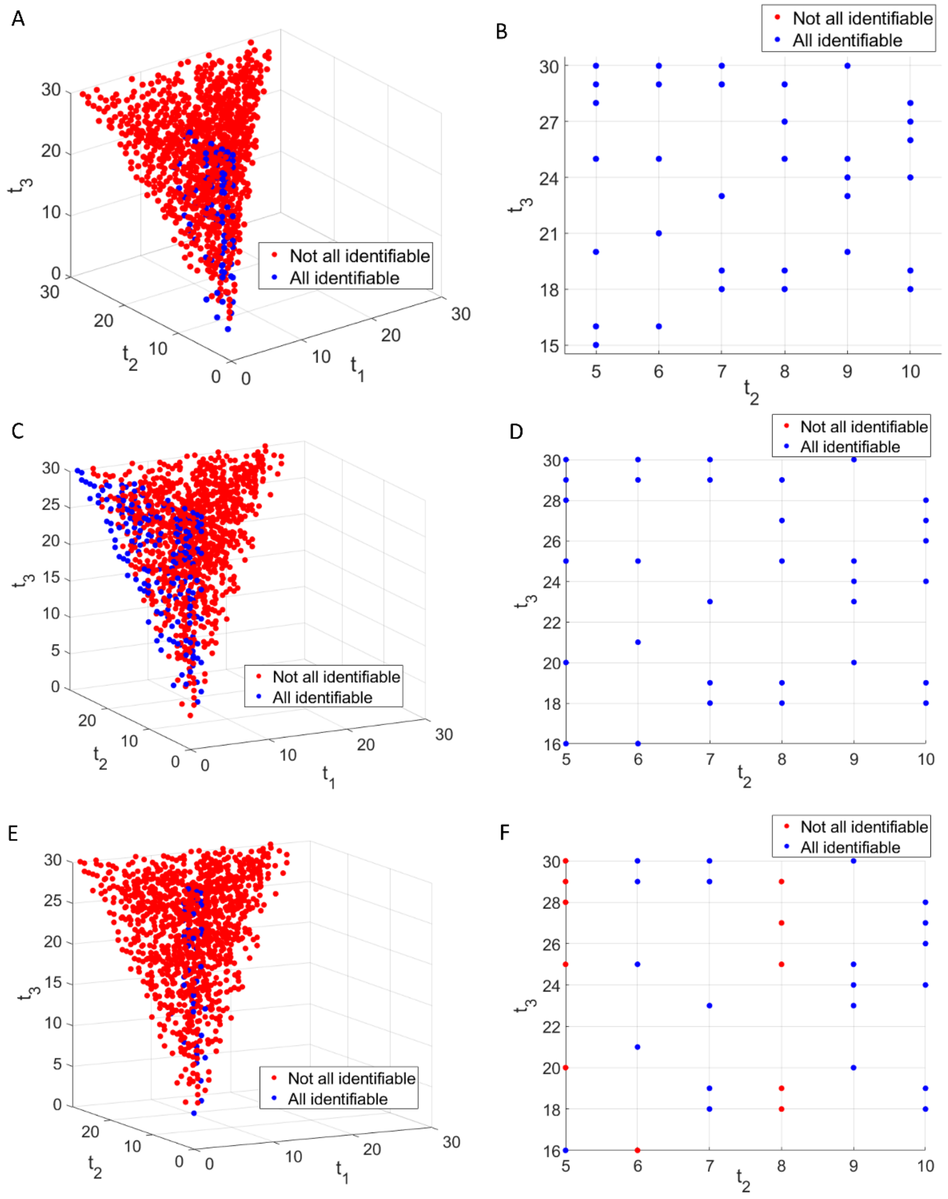

Supplementary Figure 5: Exploring robustness of the experimental design recommendation to parameter distribution shape and standard deviation. Top row: using a lognormal distribution with the same mean and standard deviation as the default normal distribution. (A) shows the classification of a random sampling of 3-day protocols (t_1, t_2, t_3) by whether they result in both parameters being practically identifiable (blue circles) or not (red circles). (B) only shows the protocols found in the recommended protocol design region P (determined using the default value of σ). Middle row: using a normal distribution with the same mean as the default normal distribution, but a 25% smaller standard deviation. (C) shows the classification of a random sampling of 3-day protocols by whether they result in both parameters being practically identifiable or not. (D) only shows protocols found in P. Top row: using a normal distribution with the same mean as the default normal distribution, but a 25% larger standard deviation. (E) shows the classification of a random sampling of 3-day protocols by whether they result in both parameters being practically identifiable or not. (F) only shows protocols found in P.

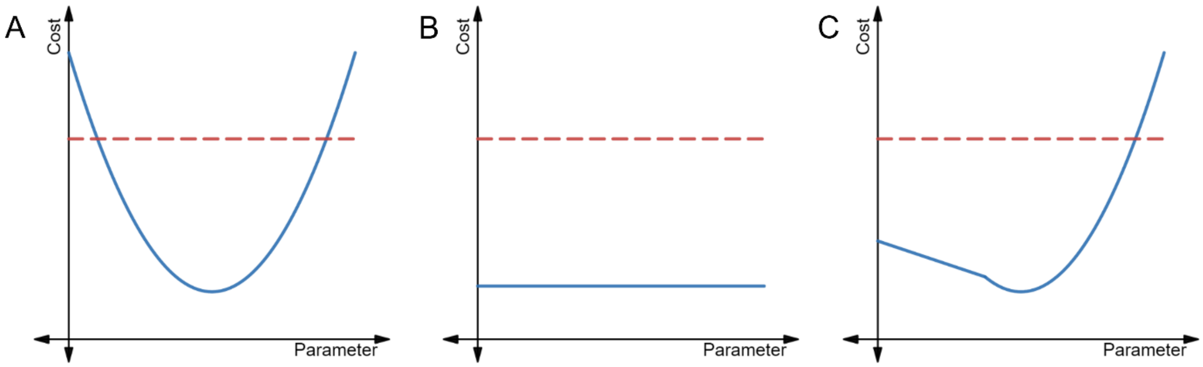

Supplementary Figure 6: Illustration of the profile likelihood method. Profile likelihood curves (blue) of (A) a practically identifiable parameter, (B) a structurally non-identifiable parameter, and (C) a structurally identifiable but practically non-identifiable parameter. Thresholds for the 95% confidence intervals are indicated with red dashed lines. The figure is adapted from “A practical guide for the generation of model-based virtual clinical trials” (M. Craig, J.L. Gevertz, I. Kareva and K.P. Wilkie, 2023. *Frontiers in Systems Biology* **3**: 1174647).
